# Salivary biglycan-neo-epitope-BGN^262^: A novel surrogate biomarker for equine osteoarthritic sub-chondral bone sclerosis and to monitor the effect of short-term training and surface arena

**DOI:** 10.1016/j.ocarto.2023.100354

**Published:** 2023-03-15

**Authors:** S. Adepu, M. Lord, Z. Hugoh, S. Nyström, L. Mattsson-Hulten, K. Abrahamsson-Aurell, C. Lützelschwab, E. Skiöldebrand

**Affiliations:** a Department of Pathology, Institute of Biomedical Sciences and Veterinary Public Health, Swedish University of Agricultural Sciences, Uppsala, Sweden; b Department of Physics, Chalmers University of Technology, Gothenburg, Sweden; c Department of Clinical Chemistry and Transfusion Medicine, Institute of Biomedicine, Sahlgrenska University Hospital, Gothenburg University, Gothenburg, Sweden; d Hallands Djursjukhus Kungsbacka Hästklinik, Älvsåkers Byväg 20, 434 95 Kungsbacka, Sweden

**Keywords:** Biglycan neo-epitope, Osteoarthritis, Saliva, Training, Riding surface

## Abstract

**Objective:**

We aimed to delineate a novel soluble Biglycan Neo-epitope-BGN262 in saliva from young reference and osteoarthritic horses in conjunction with the influence of short-term training exercise, riding surface hardness, circadian rhythm, and feeding on its soluble levels.

**Design:**

A custom-made inhibition ELISA was used for the quantification of BGN262 in saliva. Cohort 1: A cross-sectional study comprising reference (N ​= ​19) and OA horses (N ​= ​9) with radiographically classified subchondral bone sclerosis. Receiver operating characteristic curve analysis was performed to evaluate the robustness of BGN262. Cohorts 2 (N ​= ​5) & 3 (N ​= ​7): Longitudinal studies of sampling during a short-term training exercise (sand-fibre) and a cross-over design of short-training exercise on 2 different riding arenas (sand and sand-fibre), respectively. Capillary western immunoassay was used to determine the BGN262 molecular size in a selection of saliva samples collected from cohort 1.

**Results:**

Cohort 1: Salivary BGN262 levels were significantly higher in the OA group. The Receiver operating characteristic curve analysis showed an area under the curve of 0.8304 [0.6386 to 1.022], indicating a good separation from the reference group. Cohorts 2 & 3: Salivary BGN262 levels significantly changed during the exercise on sand and sand-fibre arena, with a trend towards higher levels for sand-fibre. The size of the BGN262 fragment determined by Capillary western assay was 18 ​kDa.

**Conclusions:**

The data presented show saliva BGN262 levels as a novel biomarker in evaluating the influence of exercise, and interaction with riding arenas alongside assessing osteoarthritis severity.

## 1 Introduction

Osteoarthritis (OA) pathogenesis is multifactorial however especially in weight-bearing joints, it is often mechanically driven in both humans and horses [1,2]. OA is a leading cause of early retirement in athletic horses, which is mainly induced by joint overload as a consequence of intense training at an early age [3,4].

The early OA progression is mostly asymptomatic. The associated low-inflammation activates extracellular matrix (ECM) degradation, and intensifies bone-cartilage unit crosstalk that plays a crucial role in disease manifestation [5,6]. Biglycan (BGN), a proteoglycan found to be expressed in both skeletal and non-skeletal tissues [7]. Although BGN is found in both cartilage and bone, its role is more prominent in bone structure, formation, bone matrix mineralization, thereby maintaining homeostasis [[8], [9], [10], [11]]. The soluble BGN was documented to increase in SF from patients with OA and RA and BGN neo-epitope i.e., serum BGM (cleavage site 344′YWEVQPATFR) in a collagen-induced RA mouse model [12,13]. In our recent work the BGN neo-epitope i.e., BGN^262^ (cleavage site ^262^GLGHNQIRM) levels in SF were proportional to the long-term training period in racehorses, the severity of subchondral bone sclerosis (SCBS) and the presence of chip fractures [14]. Interestingly, BGN degradation, evident by the BGN^262^ increase in young racehorses as early as during the first 6-month interval of training, mirrors the response to the joint load. Reducing the mechanical stress on the joint before the onset of clinical symptoms can minimise the risk of further disease progression to a greater extent.

Several reports are linking the injury incidences to the training surfaces [15,16]. The riding surface properties (hardness, compactness, grip, uniformity) can negatively impact horse welfare and performance [17].

For several years, biomarkers have been considered a diagnostic tool for the early detection of OA, with specific biomarkers indicating specific stages in disease manifestation [[18], [19], [20]]. Serum and SF sampling are invasive and to some extent complex, limiting their use as monitoring tool for disease progression. Changes in urinary glycosaminoglycan has been reported to be associated with age, training and OA in horses, but although, urine sampling could be non-invasive, it cannot be planned unless catheterization is involved [21]. Instead, saliva could be an ideal sample for screening as it is fairly simple to collect, contains many molecules that are otherwise present in SF and serum, and its composition reflects the physiological and disease state of the body [[22], [23], [24]].

The objective of this study was to detect and quantify the specific biomarker, soluble BGN^262^, in the saliva of reference horses and OA horses with defined radiological changes, and to determine the impact of short-term training exercise, surface arena hardness, circadian rhythm and feeding on its concentrations.

## 2 Material and methods

### 2.1 Horse material

The saliva samples were collected from four different horse cohorts. The ethical permission number for the studies performed is 5.8.18–02896/2018. For Cohorts 3 ​all the owners have signed a written informed consent form for their horse to be included in the study. Table 1 in supplementary data details age, gender and breed for all the cohorts.

**Table 1:** BGN^262^ concentration in saliva.

Cohort	BGN^262^conc (ng/ml)
1.a (*N* ​= ​9)	17.93 ​± ​7.49 [12.17–23.69]
1.b ​(*N* ​= ​19)	11.09 ​± ​3.03 [9.63–12.55]
2	
Sand-fiber arena	
TP1 (*N* ​= ​5)	13.67 [6.97–20.38]
TP2 (*N* ​= ​5)	21.77 [16.64–26.90]
TP3 (*N* ​= ​5)	24.41 [18.58–30.24]
TP4 (*N* ​= ​5)	24.01 [13.72–34.30]
TP5 (*N* ​= ​4)	11.51 [7.84–15.17]
3	
Sand arena	
TP1 (*N* ​= ​7)	14.11 [11.39–16.84]
TP2 (*N* ​= ​7)	20.64 [13.36–27.92]
TP3 (*N* ​= ​7)	26.83 [16.91–36.75]
TP4 (*N* ​= ​7)	23.87 [13.36–34.39]
TP5 (*N* ​= ​7)	18.75 [12.22–25.28]
Sand-fiber arena	
TP1 (*N* ​= ​7)	14.59 ​± ​3.9 [10.9–18.2]
TP2 (*N* ​= ​7)	25.93 ​± ​10.9 [15.7–36.0]
TP3 (*N* ​= ​7)	35.76 ​± ​17.5 [19.5–52.0]
TP4 (*N* ​= ​7)	33.90 ​± ​15.0 [19.9–47.8]
TP5 (*N* ​= ​7)	15.61 ​± ​5.2 [10.7–20.4]

Showing the BGN^262^ concentrations as ng/ml for cohorts 1, 2 and 3

The data is presented as mean with 95% [CI].

N ​= ​number of horses, TP ​= ​time points.

#### 2.1.1 Cohort 1

Horses with OA (cohort 1.a) were recruited from Hallands Djursjukhus, Kungsbacka Horse clinic, Sweden (*N* ​= ​5) and University Animal Hospital (UDS), Uppsala, Sweden (*N* ​= ​4). The recruited horses showed clinical lameness by the reaction to the flexion test and were diagnosed with OA by radiological changes in the joint or ultrasound. None of the horses was treated with corticosteroids within the three months before saliva collection. The reference group comprises saliva collected from Standardbred trotters (*N* ​= ​19) aged 1.5 years (cohort 1.b), trained by the same professional trainer. The horses were entered into a long-term training program just a month before saliva collection, followed by a training program with a slow trot distance of 2 ​km, a maximum of four days per week.

#### 2.1.2 Cohort 2

A short-term training study was performed on riding horses (*N* ​= ​5), (private owned) housed at the same stable in Gothenburg, Sweden. The saliva collection was carried out according to the following scheme (Time points: TP): TP1) In the stable at rest i.e., 1 ​h pre-training, TP2) 30 ​min post warmup (15 ​min free-walk and 15 ​min walk, trot and canter), TP3) 20 ​min post-training (intensive workout with increased collection in all gaits) TP4) 15 ​min post cool down (5 ​min trot and 10 ​min free-walk) and TP5) 1 ​h post-training respectively. The total intense interval was 20 ​min. The total warm-up and cool-down times were 45 ​min.

#### 2.1.3 Cohort 3

A short-term training study was performed on riding horses (*N* ​= ​7), (private owned) recruited from Ida farm, Wellington, US. The horses were trained on two different surfaces in a crossover design: a) sand (CapillaryFlow-Wellington, FL,USA) and b) sand-fibre. The Orono biomechanical surface tester (OBST) has been used to measure the surface of the tracks in vertical and horizontal directions, for impact firmness, cushioning, responsiveness, grip and uniformity and were graded accordingly [25].The mean number of measured drop sites for all variables per arena was 15. The saliva collection was carried out according to the following scheme: TP1) In the stable at rest i.e., 1 ​h pre-training, TP2) 10 ​min post warmup (5 ​min free-walk and 5 ​min walk, trot and canter), TP3) 20 ​min post-training (intensive workout with increased collection in all gaits) TP4) 5 ​min post cool-down (free walk) and TP5) 1 ​h post-training respectively. The total intense interval was 20 ​min. The total warm-up and cool-down times were 15 ​min.

#### 2.1.4 Cohort 4

Saliva was collected from horses (*N* ​= ​5) at the Swedish University of Agricultural Sciences, Uppsala, Sweden. The samples were collected at time points: TP1) 1 ​h before and TP2) 15 ​min, TP3) 30 ​min into the feeding and TP4) 1 ​h after being fed concentrated feed and hay. An additional sample TP5) was collected 1 ​h after the horse had finished their meal.

#### 2.1.5 Cohort 5

Saliva was collected from horses (*N* ​= ​5) at the Swedish University of Agricultural Sciences, Uppsala, Sweden. The samples were collected every second hour for 24 ​h to determine the circadian effect on BGN^262^ saliva levels. The first saliva sample was collected at 09:00 in the morning and the last sample was collected at 09:00 in the morning the day after.

### 2.2 Saliva collection and preparation

The saliva collection and preparation has been described in details in supplementary data.

### 2.3 BGN^262^ immunoassay

The inhibitory ELISA used has been previously developed, described and used for the detection of BGN^262^ in horse SF [14]. The ELISA method has been validated in horse saliva for intra- and inter-assay variability and linearity. The linearity was checked on three individual saliva samples. Commercial equine serum (Håtuna lab AB, Håtunaholm, Sweden) was used as an internal control to check for intra and inter-assay variation. All measurements were performed in duplicates.

### 2.4 Protein determination of saliva samples

The total protein concentration of collected saliva samples from cohorts 1, 2, 3, 4 and 5 (*N* ​= ​208) was determined with Pierce™BCA Protein Assay Kit (Thermo Scientifc™) according to the manufacturer's instructions using bovine serum albumin as a standard. Absorbance was measured at 560 ​nm by the absorbance microplate reader Infinite® F50 with Magellan™tracker software from Tecan.

### 2.5 Capillary Western blot of BGN ^262^ fragment in saliva

A selected representation (N ​= ​4) of saliva samples from cohort 1.a and cohort 1.b were analysed for detection of the BGN^262^ fragment with Wes. Capillary Western blot analysis was performed using the simple western system Wes™ (ProteinSimple), using a 2–40 ​kDa separation module with an anti-rabbit detection module (Protein simple). The analysis and preparations were performed according to the kit's protocol. The protocol is described in detail in supplementary methods.

### 2.6 Sampling of equine oral mucosal keratinocytes and immunohistochemistry

Oral mucosa cells were sampled from selected OA (N ​= ​6) and reference (N ​= ​13) horses from cohort 1. Twenty keratinocytes per sample were assessed for BGN^262^ cytoplasmic staining.

Equine OMKs were collected using Cytobrush Plus GT (Medscan, CooperSurgical) from the horse underlip and smeared on TOMO Adhesion Microscope Slides (10000–038, Avantor) and dried RT for 1–3 ​h. The glass slides were fixated and stained for IHC (Suppl. material).

### 2.7 Statistical analysis

Descriptive analyses are presented with mean and confidence intervals for the means [CI]. Shapiro-Wilks normality tests, made independently on each cohort, do not support a deviation from the lognormal distribution for BGN^262^ for cohort 1, 2, 4, 5. Hence, logarithmic values for BGN^262^ were used in significance tests using both parametric and non-parametric methods. For cohort 3 the Shapiro-Wilks normality tests imply the data as normally distributed hence the BGN^262^ values are not log transformed.

For cohorts 1 and 1a, where horses with OA were compared to the reference group, comparisons were made using t-tests and Wilcoxon rank-sum tests. For cohorts 2, 4 and 5, *i.e.* exercise, diurnal and feeding cohorts, we treated time as an ordinal variable and performed a one-way ANOVA test on the logarithmic BGN^262^ data. Since cohort 3 data is normally distributed a one-way ANOVA followed by post-hoc test using Bonferroni correction were performed to test for the significant differences between the time points. The test indicated whether mean values are equal for all time points under the assumption of a common standard deviation. Additionally for cohort 3 cross-over design a paired *t*-test has been performed to test for the mean differences in BGN^262^ concentrations on two different surface arenas.

The correlation between protein concentration and BGN^262^ in saliva was tested using cohorts 1, 2, 3, 4 and 5, where both variables are measured. Tests were conducted using both Pearson and Spearman correlation coefficients.

Statistical significance was set at p ​< ​0.05. One asterisk (∗) if p ​< ​0.05; two (∗∗) if p ​< ​0.01; three (∗∗∗) if p ​< ​0.001 and four (∗∗∗∗) if p ​< ​0.0001. The statistical analysis software R (https://www.r-project.org/) version 4.1.2 was used for the analysis.

## 3 Results

### 3.1 BGN^262^ ELISA

The control serum was run in duplicates in each plate with the average BGN^262^concentration of 920 ​± ​605 ​ng/ml with an inter-assay CV of 11.37%. On the linearity test, the saliva samples showed a good recovery between 80 and 120% (Table 2, suppl. material).

#### 3.1.1 Cohort 1

**Fig. 1:**
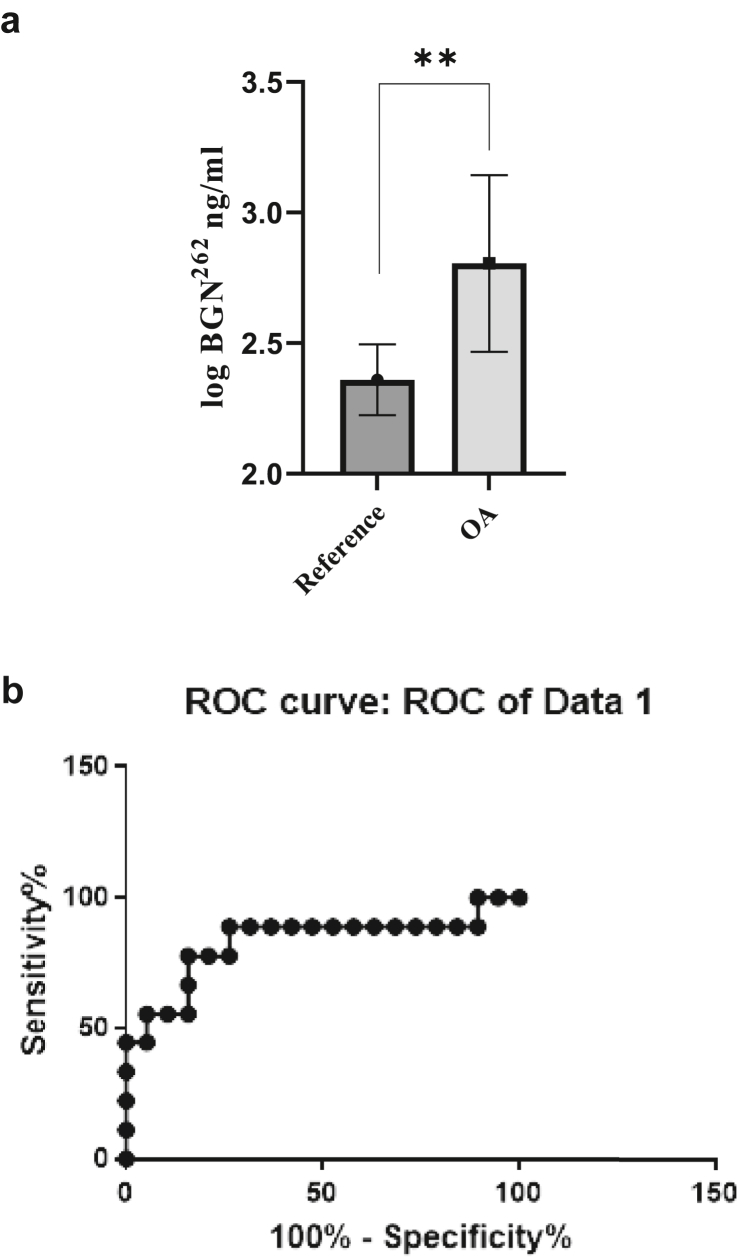
(a) (Cohort 1) the data are shown as logBGN^262^ (SEM). OA horses with radiographic changes showed a significantly increased concentration of the BGN^262^ compared to reference horses (*t*-test p ​= ​0.01962 and Wilcoxon p ​= ​0.0196) (b) (Cohort 1) Receiver Operating Characteristic (ROC) curve analysis was performed. The area under the curve was used to determine the specificity and sensitivity of the ELISA and how well the assay can distinguish between samples from Cohort 1.a (osteoarthritis with radiological changes) and Cohort 1.b (reference horses). The area under the ROC curve (AUC) was 0.8304 [0.6386 to 1.022] (p ​= ​0.0054), indicating a good separation of saliva BGN^262^ concentrations between the two groups.

The saliva BGN^262^ concentration (Table 1) was significantly higher in OA with defined radiological changes (cohort 1.a; 17.93 ​± ​5.76 [12.17–23.69]) compared to reference horses (cohort 1.b; 11.09± ​1.45 [9.63–12.55]) with p ​< ​0.01 with *t*-test and p ​< ​0.01 with Wilcoxon (Fig. 1a). The ROC analysis showed an AUC of 0.8304 [C·I 0.6386 to 1.022], (p ​< ​0.005) indicating a good separation between reference and OA horses (Fig. 1b).

#### 3.1.2 Cohort 2

**Fig. 2:**
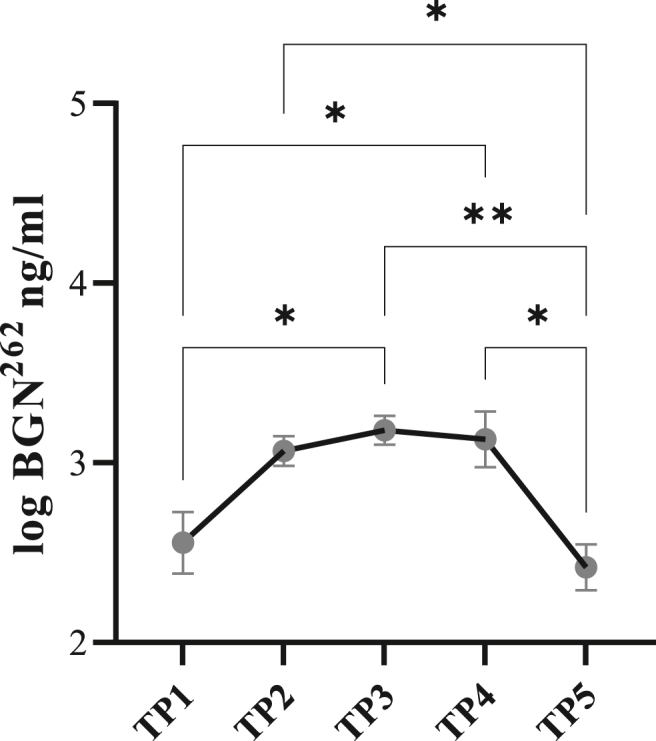
Sand-fiber arena (Cohort 2) Data were shown as logBGN^262^ (SEM) concentration. BGN^262^ concentration increase during the exercise was significant (ANOVA: p ​= ​0.001) and significantly differs between time points: TP1 vs TP3; TP1 vs TP4; TP2 vs TP5; TP3 vs TP5; TP4 vs TP5. TP1 ​= ​1 ​h before exercise, TP2 ​= ​30 ​min of warmup, TP3 ​= ​20 ​min of extensive training, TP4 ​= ​15 ​min of cool down, TP5 ​= ​1 ​h after exercise.

At baseline i.e., TP1 the saliva BGN^262^ concentration was 13.7± ​5.4 [6.70–20.37]. Already after 30 ​min of warm-up (TP2) the levels increased and continued to stay high after intense exercise and cool-down (TP3 and TP4, respectively), with the maximum concentrations in saliva found at TP3 (24.4 ​± ​4.7 [18.57–30.23]). All values returned to baseline at TP5 (11.5 ​± ​2.9 [7.84–15.17]). The change in the saliva BGN^262^ concentration during the short-term training exercise study was statistically significant. (ANOVA p ​< ​0.001) with significant differences between multiple time points: TP1 vs TP3; T1 vs T4; T2 vs T5; T3 vs T5; T4 vs T5 (Fig. 2. Table 1).

#### 3.1.3 Cohort 3

The OBST evaluation of the two riding surfaces showed major differences in impact firmness and cushioning. For the sand-fibre arena, the impact firmness and cushioning were graded harder and more compact than the sand arena surface. The other parameters (responsiveness, grip and uniformity) were similar for both arenas (suppl.material Fig. 5a and b). For the cross-over design there was a trend towards higher BGN^262^ values on sand-fibre arena compared to sand arena however the increase did not reach the statistical significance (p ​= ​0.09).

**Fig. 3:**
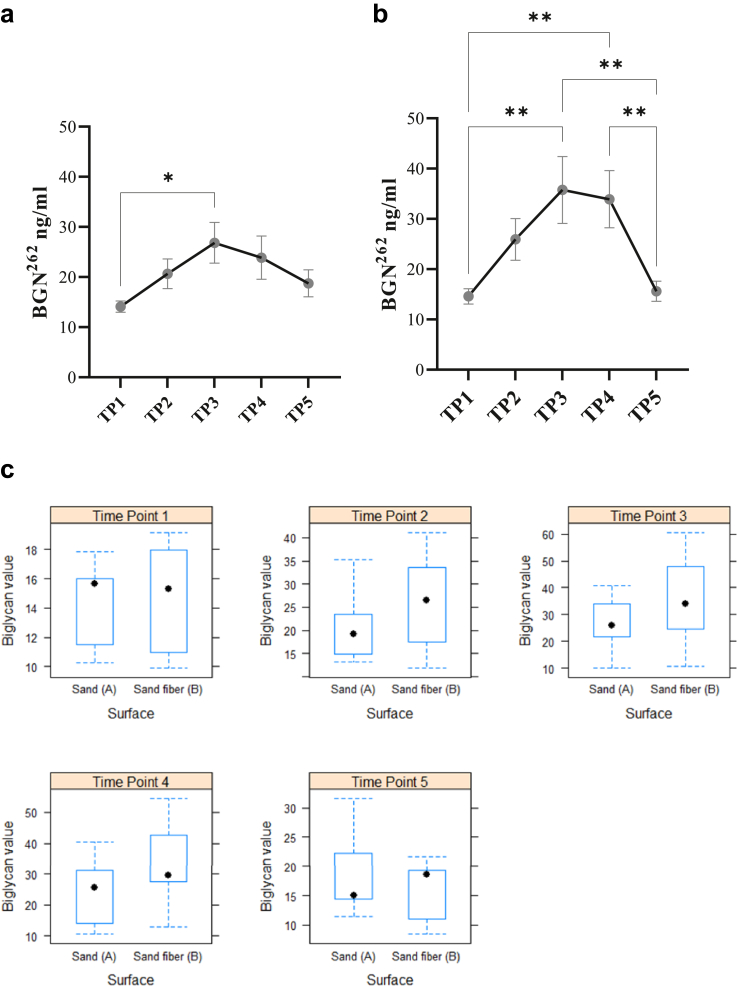
(a). Sand arena (b). Sand-fiber arena (Cohort 3) Data were shown as BGN^262^ (SEM) concentration. 3a. Sand arena - BGN^262^ concentration increase during the exercise was significant (ANOVA: p ​= ​0.017) and significantly differs between time points; TP1 vs TP3 3b. Sand-fibre arena - BGN^262^ concentration increase during the exercise was significant (ANOVA: p ​= ​0.001) and significantly differs between time points: TP1 vs TP3; TP1 vs TP4; TP2 vs TP5; TP3 vs TP5; TP4 vs TP5. TP1 ​= ​1 ​h before exercise, TP2 ​= ​10 ​min of warmup, TP3 ​= ​20 ​min of extensive training, TP4 ​= ​5 ​min of cool down, TP5 ​= ​1 ​h after exercise. (c). Box plots – Sand arena vs Sand-fiber arena (c) (Cohort 3) Data were shown as mean of BGN^262^ concentration with min and max for each timepoint on sand arena and sand-fiber arena.TP1 ​= ​1 ​h before exercise, TP2 ​= ​10 ​min of warmup, TP3 ​= ​20 ​min of extensive training, TP4 ​= ​5 ​min of cool down, TP5 ​= ​1 ​h after exercise.

Within each group (sand and sand-fibre arena) BGN^262^ concentration changed significantly during the exercise (ANOVA: p ​= ​0.013 & 0.003). Bonferroni post-hoc test resulted in significant differences between time points; TP1 vs TP3 on sand whereas on sand-fibre there are significant differences between multiple time points: TP1 vs TP3; TP1 vs TP4; TP2 vs TP5; TP3 vs TP5; TP4 vs TP5 respectively (Fig. 3a & b; Table 1) Box-plots shows the mean difference in values at each time point between sand arena and sand-fibre arena (Fig. 3c).

#### 3.1.4 Cohort 4

The saliva BGN^262^ concentration was 8.7± ​2.6 [5.45–11.95] before feeding and did not change during and after feeding. The data are presented in suppl. material Fig.1 & Table 3.

#### 3.1.5 Cohort 5

The saliva BGN^262^ concentration did not change diurnally. The values are presented suppl. material Fig.2 & Table 4.

### 3.2 Protein determination and correlation to BGN^262^ fragment in saliva

There was no significant correlation between the total protein concentration and the saliva BGN^262^ concentration. When protein and the BGN^262^ concentrations were analysed separately within the groups (for all the cohorts), no correlations were found (Suppl.data Tables 5 and 6).

### 3.3 Determination of molecular weight of BGN^262^ fragment in saliva

**Fig. 4:**
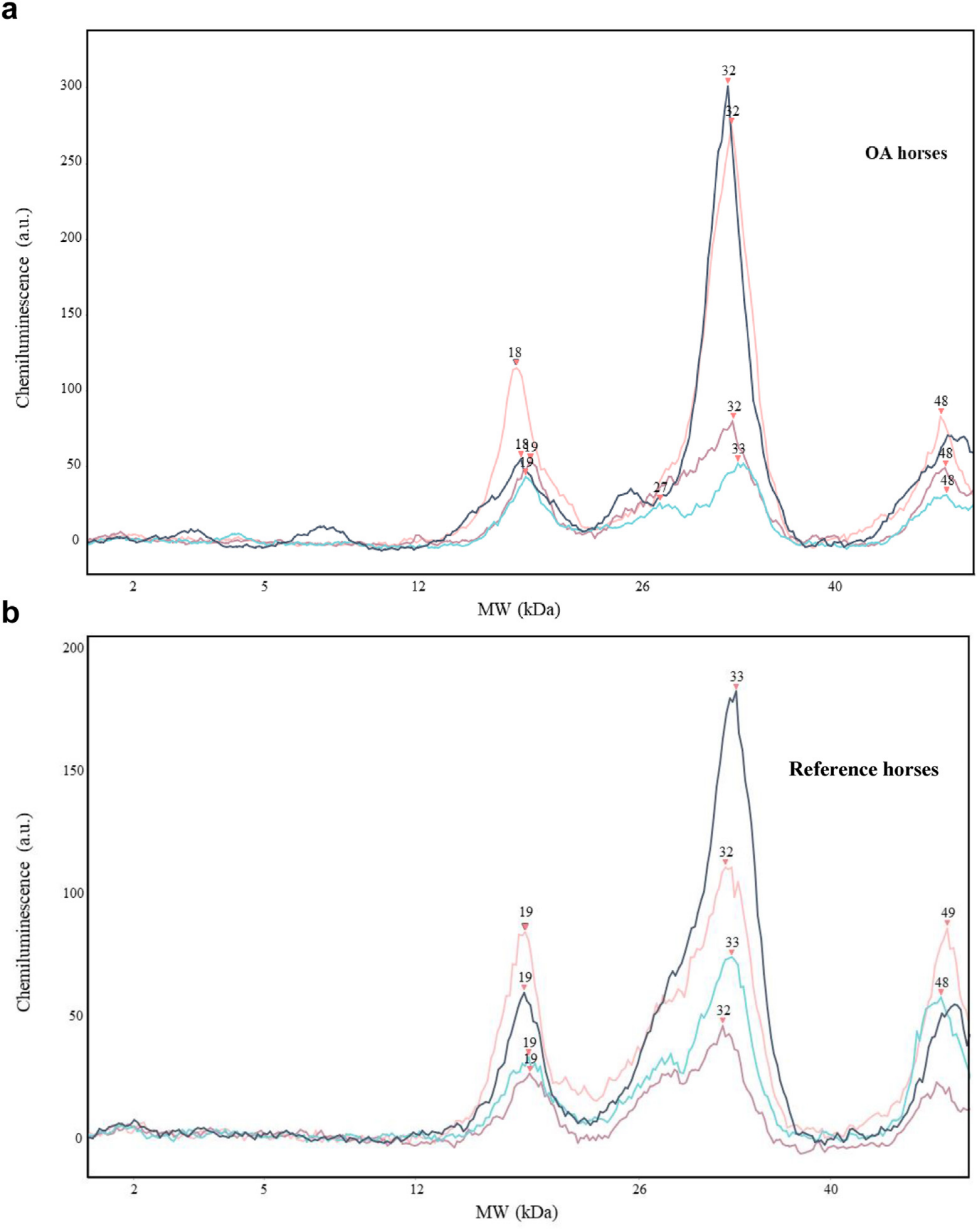
(a) Electropherogram with a selection of OA horses i.e., Cohort 1.a (*N ​= ​4*) analysed with the anti-BGN^262^ monoclonal antibody diluted 1:50 and 1 ​mg/ml of saliva. The specific peak of 18–19 ​kDa corresponds to the BGN^262^). The peaks at approximate 32–33 and 48 ​kDa, corresponds to IgG light & heavy chain (Suppl. material Fig 3a.).The detected chemiluminescence is shown as a function of apparent molecular weight (MW). Chemiluminescence is expressed as an arbitrary unit (a.u.). (b) Electropherogram with a selection of reference horses i.e., Cohort 1.b ​(*N ​= ​4*) analysed with the anti-BGN^262^ monoclonal antibody diluted 1:50 and 1 ​mg/ml of saliva. The specific peak of 18–19 ​kDa corresponds to the BGN^262^). The peaks at approximate 32–33 and 48 ​kDa, corresponds to IgG light & heavy chain (Suppl. material Fig 3a.).The detected chemiluminescence is shown as a function of apparent molecular weight (MW). Chemiluminescence is expressed as an arbitrary unit (a.u.).

The Wes detected a specific peak of 18–19 ​kDa corresponding to the BGN^262^ fragment in the saliva from both OA and reference horses (Table 2 & Fig. 4a and b). An additional peaks at approximate MW of 32–33 and 47–48 ​kDa (Suppl. material Fig. 3a.) were detected as a result of cross-reactivity of anti-BGN^262^ Mab with horse IgG light and heavy chain. This was confirmed by horse IgG-specific antibody also giving the same result with peak at 32 ​kDa and a small peak at 48 ​kDa (Suppl. material Fig. 3a). The anti-albumin antibody did not detect any peaks within the desired range (Fig. 3b.). Intra-system interaction with antibody specificity for anti-BGN^262^ Mab was performed according to kit instructions, where no unspecific binding could be found with appropriate controls i.e., no primary antibody, no secondary nor using sample buffer as protein load instead of saliva. The antibody specificity was assessed where none of the controls showed non-specific binding (Suppl. material Fig. 3c.).

**Table 2:** The molecular weight and SN-values for the peaks of interest detected with Capillary western Immuno assay (Wes).

	n	Molecular Weight (kDa)		Signal-to-noise	
		Mean ​± ​SD	Min-max	Mean ​± ​SD	Min-max
Cohort 1.a (OA horses)	4	18.5 ​± ​0.58	18–19	32.33 ​± ​18.14	19.8–58.7
Cohort 1.b (reference horses)	4	19.0 ​± ​0	19	22.55 ​± ​9.49	13.1–32.5

The saliva from OA & reference horses showing the mean BGN^262^ peaks at a molecular weight of 18.5–19 kD. A peak with the signal-to-noise ratio (SN) above 10 is considered as a valid peak.

In saliva a specific peak was found at 18–19 ​kDa with signal-to-noise ratio (SN) 27.44 ∗ 14.39, as a peak as well as a peak at 32–33 ​kDa with SN 65.70 ∗ 43.50, which overlapped with the molecular weight of the IgG light chain. Both peaks were detected in horses from cohort 1a (*N* ​= ​4) and cohort 1b (*N* ​= ​4). Unfortunately, saliva samples from all horses in cohort 1 could not be included due to the limited protein amount in samples.

### 3.4 Oral mucosal keratinocytes and immunohistochemistry

**Fig. 5:**
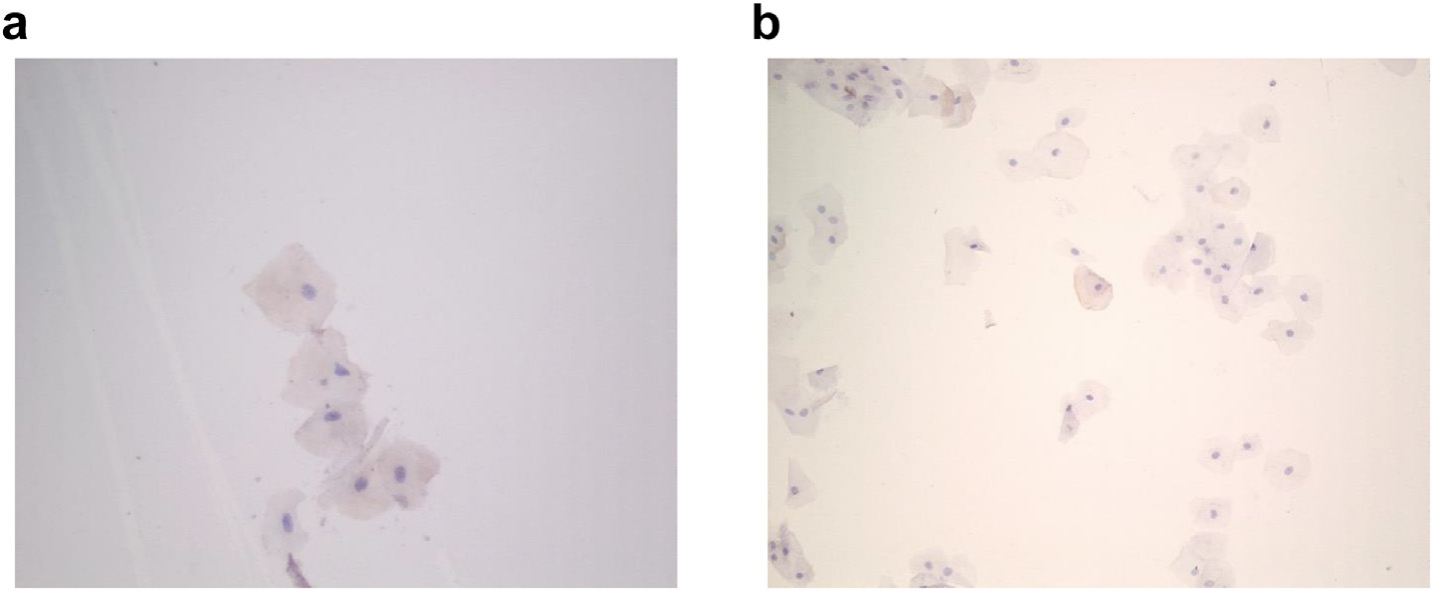
(a) reference horse keratinocytes & (b) OA horse keratinocytes- 200× images showing few cells with intracytoplasmic staining for BGN^262^ in oral keratinocytes.

Oral keratinocytes from cohort 1 both OA (4 out of 6) and reference group (10 out of 13) showed faint intracytoplasmic BGN^262^ staining (Fig. 5). None of the cells showed intra-nuclear staining (nuclear localization of BGN^262^ has been observed in OA bone cells [14].

## 4 Discussion

In the racing industry, it is essential to start race training at a young age to properly adapt their musculoskeletal system accordingly [26,27]. The age at the start and the joint health status are crucial factors, alongside other debatable factors such as the exercise type, foot-surface interaction, intensity, duration etc. All of this can have a profound effect on the adaptation process and predisposition to OA development and progression [4,28,29].

From the aspect of animal welfare and economic burden, there is a pressing demand for biomarkers that can identify early biochemical degradation of joint structures triggered by demanding training in young horses, which untended can eventually lead to OA and joint pain. Finding easy-to-use biomarker(s) that help in the preventive evaluation of horses undergoing various training regimens and in the diagnosis of incipient OA would be highly beneficial.

To the best of our knowledge, we are the first to present a novel soluble BGN neo**-**epitope in saliva for OA, equine training exercise, together with indications on the influence of riding ground surface arena. Our study shows that the presence of chronic OA (cohort 1.a) associated with elevated saliva BGN^262^ levels. This was in accordance to high SF BGN^262^ concentrations in OA horses [14]. Also, from this previous study, it was clear that the BGN^262^ levels are not age dependent but are highly sensitive to early race training [14]. Therefore, the reference group (cohort 1.b) in the current study included young horses that had been trained only for a month.

There are several reports of human and equine saliva biomarkers in the diagnosis of systemic diseases such as; diabetes mellitus, breast cancer and cardiovascular disease and infections [30,31]. Pain biomarkers such as nerve growth factor (NGF), calcitoningene-related peptide (CGRP) and glutamate were also successfully quantified in human saliva [23,24,[32], [33], [34]].

Short-term training also led to elevated BGN^262^ levels (cohort 2), another leverage is the sensitivity of saliva BGN^262^ levels (cohort 3) towards riding surface composition in terms of impact firmness and cushioning. The horses in cohorts 2 & 3 were privately owned and, based on the anamnestic interview with the owner, some of them were diagnosed with OA. This explains the wide range in baseline saliva BGN^262^ levels when compared to the reference group and the OA group (Table 1).

Several OA biomarkers in serum and SF, such as cartilage oligomeric matrix protein (COMP), collagen type I and II and aggrecan have been shown to respond to exercise in humans and horses [27,35]. Stress biomarkers such as cortisol and metabolic biomarkers have been assessed in humans and equine saliva for evaluation of the impact of training and exercise programs [[36], [37], [38]]. However, biomarkers indicating the effect of exercise on joint tissue integrity both in health and disease are lacking. In our study, horses exposed to short-term training exercise on a sand-fibre arena (cohort 2) showed an increase in BGN^262^ with a peak following the most intense exercise interval.

In cohort 3 also the short-term exercise also led to a significant increase in BGN^262^ levels on both sand arena and sand-fibre. In the crossover design there was a trend of higher increase of BGN^262^ levels on sand-fibre arena, which is more hard and compact in nature than sand-arena (Fig. 3c). The results are in agreement with other studies showing the negative impact of harder surfaces on the musculoskeletal system [15,29]. A larger sample size of 25 horses is needed (with a power of 0.8) to show a statistical significant difference for the two riding surface arenas and will be used when next study is planned.

Taken together, the health status of the joint, training intensity, and riding surface arena characteristics could all be contributing factors for the salivary BGN^262^ levels, which in turn reflects the burden and impact load on the joints. More studies are warranted in designing a safe, tailored, training regimen at an individual level.

The diagnostic potential of saliva relies on the porous capillaries surrounding the salivary glands. By passive diffusion, filtration or active transportation molecules of different sizes and charges can reach the saliva from the bloodstream. Approximately 20–30 different proteins that are detectable in the human blood can also be traced in saliva [39].

In horses, the saliva secretion occurs mainly during chewing, with increased production during eating [40]. Chewing the bit during riding is a mechanical trigger for saliva production [41]. In humans, circadian rhythm is known to influence the saliva flow volume, thereby modifying the concentrations of salivary electrolytes and proteins [42]. Similarly, in horses diurnal and seasonal variations have been shown to affect saliva cortisol, salivary alpha-amylase, total esterase, butyrylcholinesterase, adenosine deaminase, and creatinine kinase concentrations [43]. Total protein concentration did not correlate to the change in BGN^262^ levels. In our study, neither feeding (chewing) nor circadian variations affected the BGN^262^ concentrations in saliva making it a good candidate for random sample collection.

BGN is a component of oral mucosa keratinocyte (OMKs) ECM [44]. BGN has been shown to localize in differentiating keratinocytes, scarring and oral cancer [45,46], and the BGN in OMK-ECM can undergo degradation during inflammatory processes. Therefore, we investigated whether BGN^262^ could be secreted or released from these epithelial cells in the mucosa thus contributing to the saliva BGN^262^ concentration. In previous studies, the BGN^262^ staining of chondrocytes, osteocytes, and bone lining cells including osteoblasts within osteochondral lesions showed both nuclear and cytoplasmic localization. Interestingly, the nuclear localization was more pronounced in OA with increasing severity [14]. In our current study, BGN^262^ was only cytoplasmic in a few oral keratinocytes in both reference and OA horses, indicating normal turnover of the protein. IHC did not reveal any nuclear staining which we presume to be a result of pathological manifestation, together supporting the idea that the increase observed in saliva BGN^262^ levels does not originate endogenously from oral keratinocytes, instead, the increase could be a result of systemic diffusion, more specifically from SF (reflecting the joint response) and the bloodstream.

The molecular weight (MW) of BGN^262^ was identified as 18–19 ​kDa both by Wes (Fig. 4a & b) and by traditional Western blot (result not shown). The fragment was detected in the saliva of both OA with radiological changes (cohort 1.a) and reference horses (cohort 1.b). The theoretical molecular weight of BGN^262^ is 12 ​kDa. The discrepancy between theoretical and observed MW suggests that the neo-epitope from healthy and OA horses could be a result of (i) partial degradation of carbohydrate side chains attached to the core protein and/or (ii) post-translational modification (PTM). A neo-epitope arises during tissue remodelling and further undergoes different PTMs including glycosylation, citrullination, isomerization and nitrosylation as a consequence of the tissue environment. Inflamed tissues can create different PMTs than healthy tissues, however, this was not observed for BGN^262^ [47,48]. The BGN^262^ cleavage site is conserved across the species (cat, dog, pig, bovine, horse and human) and perhaps its physiological and pathological manifestation might be the same, thus making it a promising candidate to investigate in other species as well. The BGN neo-epitope with the same cleavage site (GLGHNQIR) was identified by forward and reverse degradomics resulting from the proteolytic action of HtrA1 (high temperature requirement serine protease A1) in human from both healthy and osteoarthritic knee cartilage [49].

In our study, we did not find any correlation between the saliva BGN^262^ concentrations with the total protein concentration. This is in accordance with a study in humans where saliva flow rate and total protein concentration, after mechanical stimulation did not affect the levels of CRP and myoglobin [50].

Our results are the first to show that short–term training exercise, the surface ground properties and chronic OA are all associated with a rise in soluble BGN^262^ levels. Thus, BGN^262^ levels not only serve as surrogate OA biomarker but is also highly sensitive to joint overload. The cues from soluble BGN^262^ levels can aid in identifying risk factors as well as and managing horses that are at risk of progressing into OA.

## 5 Conclusion

The possibility of non-invasive, stress-free, and easy multiple sampling of saliva enables clinicians, trainers and horse owners, to sample racehorses for preventive longitudinal monitoring of BGN^262^.

The ROC curve analysis strength of BGN^262^ and its non-alignment with feeding and circadian rhythm makes it a good biomarker candidate.

Altering the training regimens of the horse while taking into account the surface arena characteristics can help prevent OA development and progression. It is our long-term goal to quantify BGN^262^ using a validated diagnostic method such as a point-of-care tool (POC), in an accessible body fluid such as saliva, which can be used in the daily training of athletic horses.

## Author contributions

ES, ML, ZH and SA were in charge of the overall direction and planning of the study. CL and SA set up, validated the inhibition ELISA, and CL ran all the samples. SA performed the WB experiments. ML, LMH and SN performed the Wes experiments and protein determination. IHC stainings were performed by ZH. The cohorts were sampled by ZH, ML, SN, K A-A and ES.

All authors contributed to and approved the final manuscript.

## Role of the funding source

The study was funded by 10.13039/501100004359Swedish Research Council (FORMAS 2019–02069) and Svensk Djurskyddsförening.

## Declaration of competing interest

ES and LMH are among the stakeholders of SGPTH Life Science holding the patent covering the BGN^262^ neo-epitope. The other co-authors have no conflicts of interest to declare.
