# Supplementary material for: Salivary biglycan-neo-epitope-BGN262: A novel surrogate biomarker for equine osteoarthritic sub-chondral bone sclerosis and to monitor the effect of short-term training and surface arena
Source: Osteoarthr Cartil Open. 2023 Mar 15;5(2):100354. doi: 10.1016/j.ocarto.2023.100354 (PMC10033749; doi:10.1016/j.ocarto.2023.100354)
Supplement: Multimedia component 1 [file mmc1.docx]

Saliva collection and preparation

Saliva was collected using an Equisal saliva collection kit (Austin Davis Biologics Ltd) by inserting the swab through the interdental space on the horse tongue until the volume indicator changed (pink) colour. The samples were stabilised in 2 ml preservative buffer supplied in the kit, temporarily stored on ice (average time: 1hr) and moved to -20°C for a day or two before being thawed and centrifuged at 3000xg for 5 min. All samples were aliquoted and stored at -80°C until analysis.

*Immuno Histochemistry*

The glass slides were fixated with 3 % paraforlmaldehyde (PFA) for 15 minutes at room temperature (RT). The sections were washed 3 times in the wash buffer (0.05% Tween-10 mM PBS, pH 7.4) and the cells were permeabilised with 0.2 % Triton X-100 for 15 minutes followed by three times wash in the wash buffer. The endogenous peroxidase was quenched with 3% H_2_O_2_-10 mM PBS-0.05 % Tween, pH 7.4 for 5 minutes at RT. The sections were washed with distilled water followed by three times in wash buffer. All slides were incubated with 2% Normal Goat serum (Dako, Lot. 20078389, Glostrup, Denmark) for 30 minutes at RT. Immunostaining for BGN^262^ was performed at 4°C overnight with a rabbit polyclonal antibody against equine BGN^262^ (0.681 mg/mL, Lot. U8229DL260-6, Genscript) at a dilution of 1.362 µg/mL (1:4000) in 10 mM PBS, pH 7.4, and a recombinant rabbit IgG monoclonal ([EPR25A]-isotype control #ab 172730 Lot. GR3235749; Abcam, Waltham, MA, United States) was used. Staining was visualised using an HRP-conjugated anti-rabbit EnVision (Dako, CA, United States Ref. #K4003, Lot. 1199901) for 30 minutes and development with 3,3-diaminobenzidine (DAB) for 8 minutes. All sections were counterstained simultaneously with haematoxylin, dehydrated in ethanol followed by clearing in xylene and mounted with Pertex. The stained slides were imaged at 200X magnification using a bright field microscope.

*Capillary western Immuno assay (Wes)*

Saliva samples were thawed and diluted to a concentration of 1 mg/ml in 0.1x sample buffer and fluorescent master mix, containing internal standards for migration within the capillaries. The sample dilutions were vortexed and heated at 95 °C for 5 min. Following heat treatment the samples were vortexed again followed by short centrifugation and stored on ice before loading into the prefilled assay plate. The primary antibody used was the anti-BGN^262^ monoclonal antibody diluted (1:50). The anti-rabbit IgG HRP-linked antibody (#7074, Cell Signaling) diluted at (1:100) was used as secondary antibody. The denatured samples, antibodies, blocking solution, chemiluminescent substrate and wash buffer were added to the prefilled assay plate according to the protocol and inserted into the Wes, where the target protein is automatically separated based on size and estimated against a molecular weight ladder loaded into the prefilled assay plate. The Wes running protocol was run with the following parameters: sample loading time (12.6 s), stacking matrix loading time (21 s), separation time (34 min) and primary antibody exposure time (90 min). Data analysis was performed with the Compass for Simple Western software v.6.1.0, using high dynamic range 4.0 and the signal-to-noise ratio method, with noise region limited to 2-40kDa, was used.

To evaluate the antibody specificity, capillaries were run with appropriate controls (without a sample, without the primary antibody or secondary antibody). Since saliva contains several different proteins present in the blood, albumin and IgG (heavy and light chain) controls were included.

The position of the albumin peak and the IgG peak in the saliva electropherogram were assessed using a polyclonal anti-Albumin antibody (ABIN2776970, antibodies-online) and an Affinipure rabbit anti-horse IgG (H+L) (Code 308-005-003, Jackson Immuno Research), respectively.

The anti-BGN^262^ monoclonal antibody cross reactivity has been tested against purified horse IgG (Ref PEP001 LOT 155732, Biorad) at 0.015 mg/ml. The detected peaks registered with the anti-BGN^262^ antibody were accepted when the peak was not overlapping with the controls and the signal-to-noise ratio (SN) was above 10.

Table 1. Demographics of the horses - all the cohorts

|  |  | Age  mean±SD  (min-max) | Gender  (n= M, G, S) | Breed | | | | | | |  |
| --- | --- | --- | --- | --- | --- | --- | --- | --- | --- | --- | --- |
| Horses | n |  |  | SWB | STB | DWB | Pony | OH | BW | PRE/SWB | H |
| Cohort 1.a | 9 | 8.3±5.2  (1-15) | (3,5,1) | 3 | 3 | 1 | 1 | 1 |  |  |  |
| Cohort 1.b | 19 | 1.5±0 | (13,0,6) |  | 19 |  |  |  |  |  |  |
| Cohort 2 | 5 | 12.8±1.8  (10-14) | (3,2,0) | 2 |  |  | 1 | 1 |  | 1 |  |
| Cohort 3 | 8 | 9.3±3.9  (4-17) | (1,7,0) |  |  | 3 |  | 2 | 1 |  | 2 |
| Cohort 4 | 5 | 15.4±5.8  (8-24) | (5,0,0) |  | 5 |  |  |  |  |  |  |
| Cohort 5 | 5 | 16.8±3.6  (11-21) | (4,0,1) | 1 | 4 |  |  |  |  |  |  |

n: number of horses, min: minimum value, max: maximum value, M: mare, G: gelding, S: stallion, SWB: Swedish warmblood, STB: Standardbred horse, DWB: Danish warmblood, P: C pony, KWPN: Dutch warmblood, OH: Oldenburger, , PRE/SWB: Pura Raza Española/ Swedish warmblood: BW: Belgian warmblood, Hannovarian :H

Table 2- Dilution recovery of neat saliva (undiluted) and 8 dilutions in assay diluent (recovery performance between ~80-120% is considered acceptable)

|  | **Dilution factor** | **Assay values (ng/ml)** | **Expected values** | **Adjusted values (ng/ml)** | **Recovery % (neat as 100)** |
| --- | --- | --- | --- | --- | --- |
| **Sample#1** | Neat | 27,04 | 27,04 | 27,043 | 100,00 |
|  | 2 | 27,79 | 54,09 | 55,579 | 102,76 |
|  | 4 | 22,33 | 108,17 | 89,332 | 82,58 |
|  | 8 | 14,86 | 216,34 | 118,84 | 54,93 |
|  | 16 | 5,27 | 432,69 | 84,2632 | 19,47 |
|  | 32 | 1,88 | 865,38 | 60,2784 | 6,97 |
| **Sample#2** | Neat | 39,45 | 39,45 | 39,454 | 100,00 |
|  | 2 | 31,35 | 78,91 | 62,7 | 79,46 |
|  | 4 | 28,35 | 157,82 | 113,392 | 71,85 |
|  | 8 | 19,36 | 315,63 | 154,88 | 49,07 |
|  | 16 | 9,19 | 631,26 | 146,9808 | 23,28 |
|  | 32 | 3,80 | 1262,53 | 121,664 | 9,64 |
| **Sample#3** | Neat | 48,90 | 48,90 | 48,90 | 100,00 |
|  | 2 | 50,88 | 97,80 | 101,76 | 104,04 |
|  | 4 | 37,91 | 195,61 | 151,64 | 77,52 |
|  | 8 | 26,38 | 391,21 | 211,08 | 53,95 |
|  | 16 | 11,76 | 782,42 | 188,19 | 24,05 |
|  | 32 | 3,60 | 1564,85 | 115,15 | 7,36 |
|  |  |  |  |  |  |

Table 3: Saliva BGN^262^ Concentrations for Cohort 4: feeding study

| Time point | n | Mean | SD | SEM | CI [min-max] |
| --- | --- | --- | --- | --- | --- |
| TP1 | 5 | 8.71 | 2.62 | 3.25 | 5.46 – 11.96 |
| TP2 | 5 | 5.29 | 1.84 | 2.29 | 3.01 – 7.58 |
| TP3 | 5 | 4.59 | 1.83 | 2.27 | 2.32 – 6.86 |
| TP4 | 5 | 5.51 | 1.80 | 2.24 | 3.28 – 7.75 |
| TP5 | 5 | 6.66 | 2.28 | 2.83 | 3.83 – 9.49 |

Table 3:.The differences in BGN^262^ over time is not significant (p = 0.0981), one way ANOVA. The data shown as mean with SD, SEM and 95% CI.

Table 4: Saliva BGN^262^ Concentrations for cohort 5: circadian rhythm

| Time point | n | Mean | SD | SEM | CI [min-max] |
| --- | --- | --- | --- | --- | --- |
| TP1 | 5 | 17.68 | 2.63 | 3.26 | 14.42 – 20.95 |
| TP2 | 5 | 18.01 | 3.33 | 4.14 | 13.87 – 22.15 |
| TP3 | 5 | 23.62 | 8.89 | 11.04 | 12.58 – 34.66 |
| TP4 | 5 | 16.38 | 7.72 | 9.59 | 6.79 – 25.97 |
| TP5 | 5 | 20.21 | 6.34 | 7.87 | 12.34 – 28.08 |
| TP6 | 5 | 17.17 | 2.60 | 3.23 | 13.95 – 20.40 |
| TP7 | 5 | 19.23 | 7.75 | 9.62 | 9.61 – 28.85 |
| TP8 | 5 | 18.65 | 4.61 | 5.72 | 12.93 – 24.37 |
| TP9 | 5 | 20.05 | 6.66 | 8.27 | 11.78 – 28.32 |
| TP10 | 5 | 18.62 | 2.93 | 3.64 | 14.98 – 22.26 |
| TP11 | 5 | 32.61 | 18.25 | 22.66 | 9.95 – 55.27 |
| TP12 | 5 | 12.95 | 4.52 | 5.61 | 7.35 – 18.56 |
| TP13 | 5 | 19.16 | 5.95 | 7.39 | 11.76 – 26.55 |

Table 4: The difference in BGN^262^ over time is not significant (p = 0.0625), one way ANOVA. The data shown as mean with SD, SEM and 95% CI.

Table 5: Correlation estimation of total protein concentration against BGN^262^ concentration

| *N* | Type | Correlation | p-value |
| --- | --- | --- | --- |
| 208 | Pearson | --0.0632 | 0.3656 |
|  | Spearman | --0,0111 | 0.8739 |

Table 5: Showing no significant correlation between the saliva protein content and the BGN^262^ concentration in the saliva of all 5 cohorts with N= 208 (Pearson p = 0.3656 and Spearman p = 0.8739, respectively).

Table 6: Correlation estimation of protein concentration against BGN^262^ concentration

|  | *N* | Type | Correlation | p-value |
| --- | --- | --- | --- | --- |
| Cohort 1.a | 5 | Pearson | 0.62 | 0.27 |
|  |  | Spearman | 0.40 | 0.52 |
| Cohort 1.b | 19 | Pearson | -0.30 | 0.21 |
|  |  | Spearman | -0.43 | 0.07 |
| Cohort 2 | 24 | Pearson | -0.12 | 0.56 |
|  |  | Spearman | -0.05 | 0.82 |
| Cohort 3 | 65 | Pearson | 0.0339 | 0.7820 |
|  |  | Spearman | 0.1115 | 0.3607 |
| Cohort 4 | 25 | Pearson | -0.2854 | 0.1667 |
|  |  | Spearman | -0.2723 | 0.1873 |
| Cohort 5 | 65 | Pearson | -0.0572 | 0.6508 |
|  |  | Spearman | -0.0970 | 0.4410 |

Table 6: Showing no significant correlation between the saliva protein content and the BGN^262^ concentration in the saliva of individual cohorts.

Figure 1

Figure 1: (Cohort 4) BGN^262^ concentration did not show a significant increase relative to feeding time points (p=0.0981). TP1=1h before -, TP2=15 min after -, TP3=30 min after -, TP4=1h after being feed and TP5= 1h after finished meal. The data are shown as mean with SEM.

Figure 2

Figure 2: (Cohort 5) BGN^262^ concentration did not show a significant increase relative to circadian rhythm (p=0.0625). Time points (TP) represent samples taken every two hours where the first sample was collected at 09:00 and the last at 09:00 the next day. The data are shown as mean with SEM.

Figure 3:

3a.


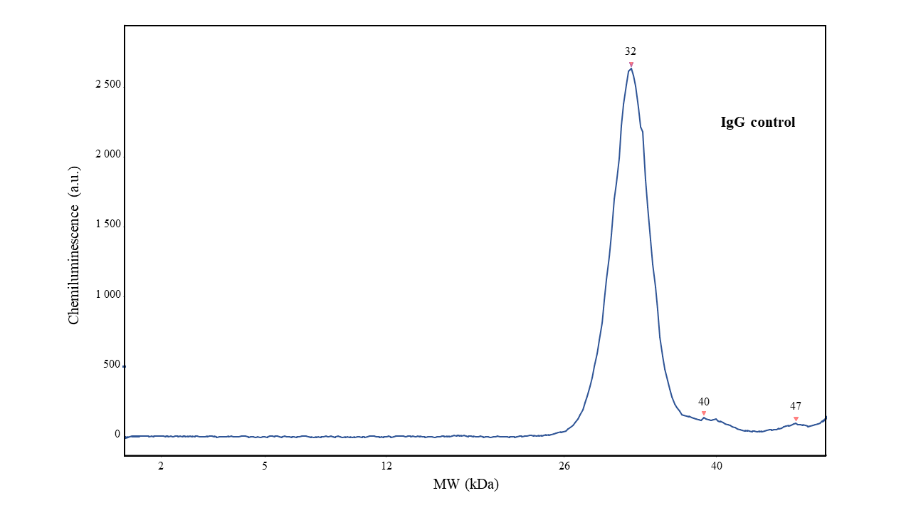


Figure 3a: Electropherogram with a representative saliva sample tested with 1:20,000 Affinipure rabbit anti-horse IgG (H+L) and 1mg/ml of saliva. The detected chemiluminescence is shown as a function of apparent molecular weight (MW). Chemiluminescence is expressed as an arbitrary unit (a.u.).

Figure 3b:


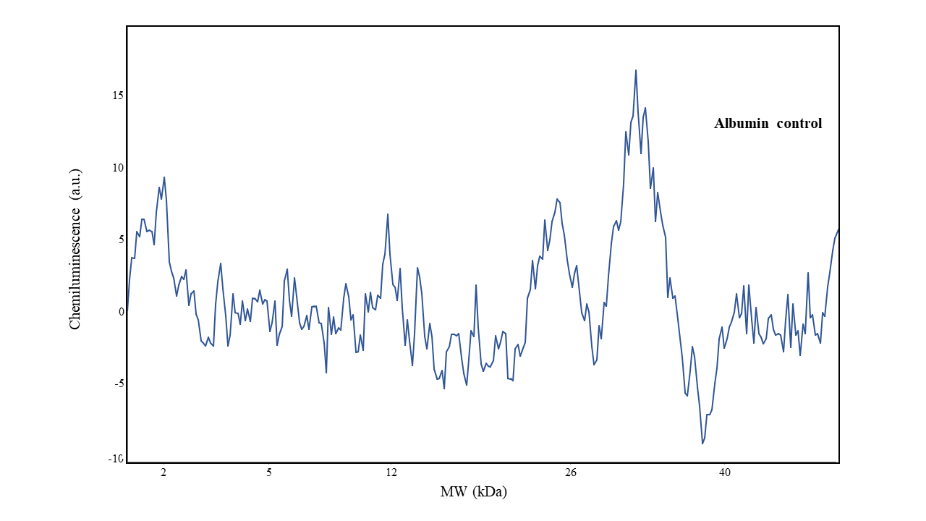


Figure 3b: Electropherogram with a representative saliva sample tested with 1:2000 polyclonal anti-Albumin antibody and 1mg/ml of saliva. The detected chemiluminescence is shown as a function of apparent molecular weight (MW). Chemiluminescence is expressed as an arbitrary unit (a.u.).

Figure 3c:


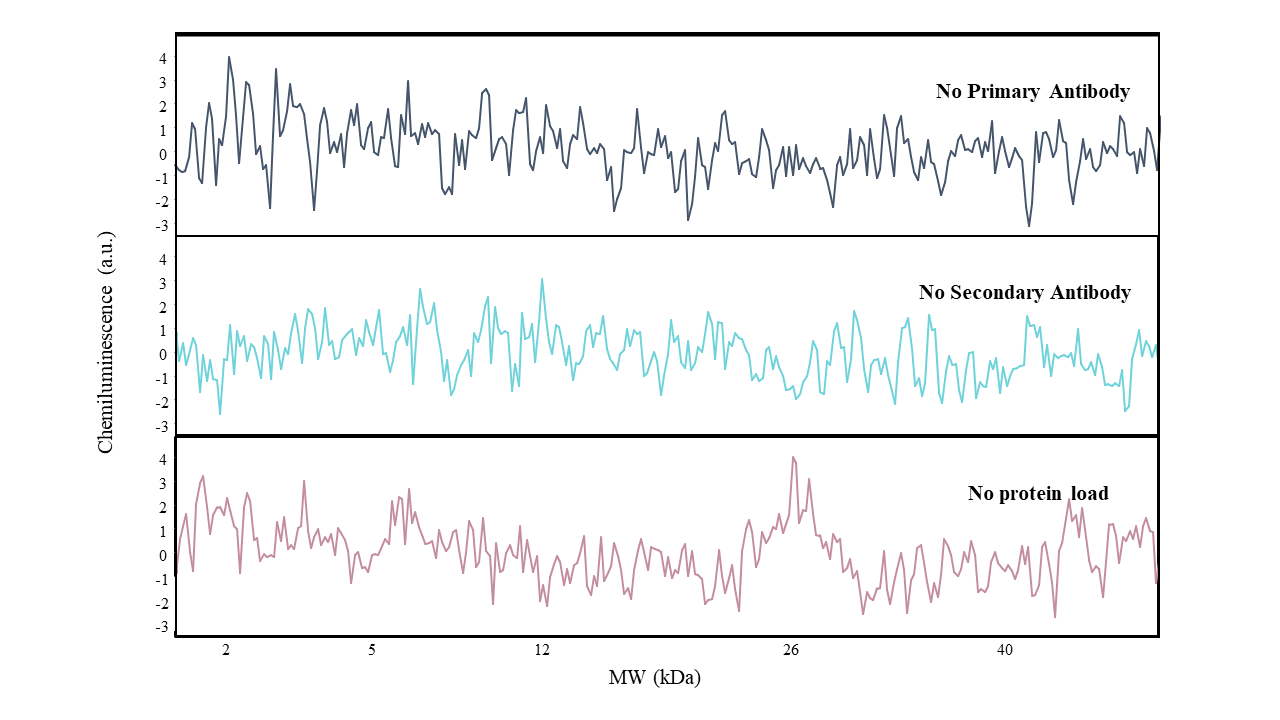


Figure 3c: Wes5: Electropherograms of controls without primary antibody, secondary antibody and without protein with no detectable peaks. Performed according to the manufacturer’s instructions. The detected chemiluminescence is shown as a function of apparent molecular weight (MW). Chemiluminescence is expressed as an arbitrary unit (a.u.).

Figure 4: The Orono biomechanical surface tester (OBST)

4a: Sand arena


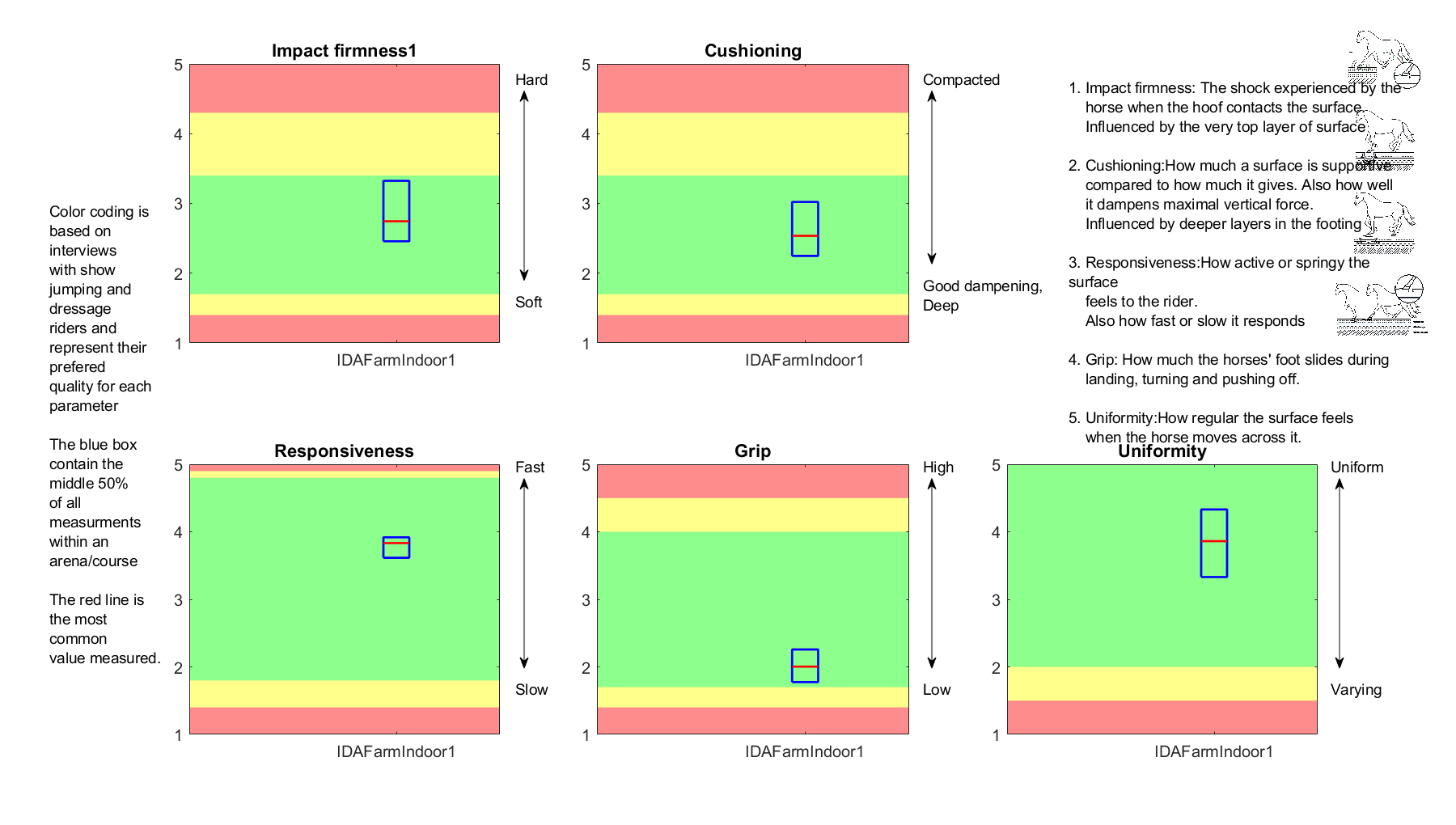


Figure 4a: The Orono biomechanical surface tester (OBST) showing the sand arena properties for impact firmness, cushioning, responsiveness, grip and uniformity. The OBST evaluation result showing ideal grade for the parameters measured.

4b: Sand-fiber arena


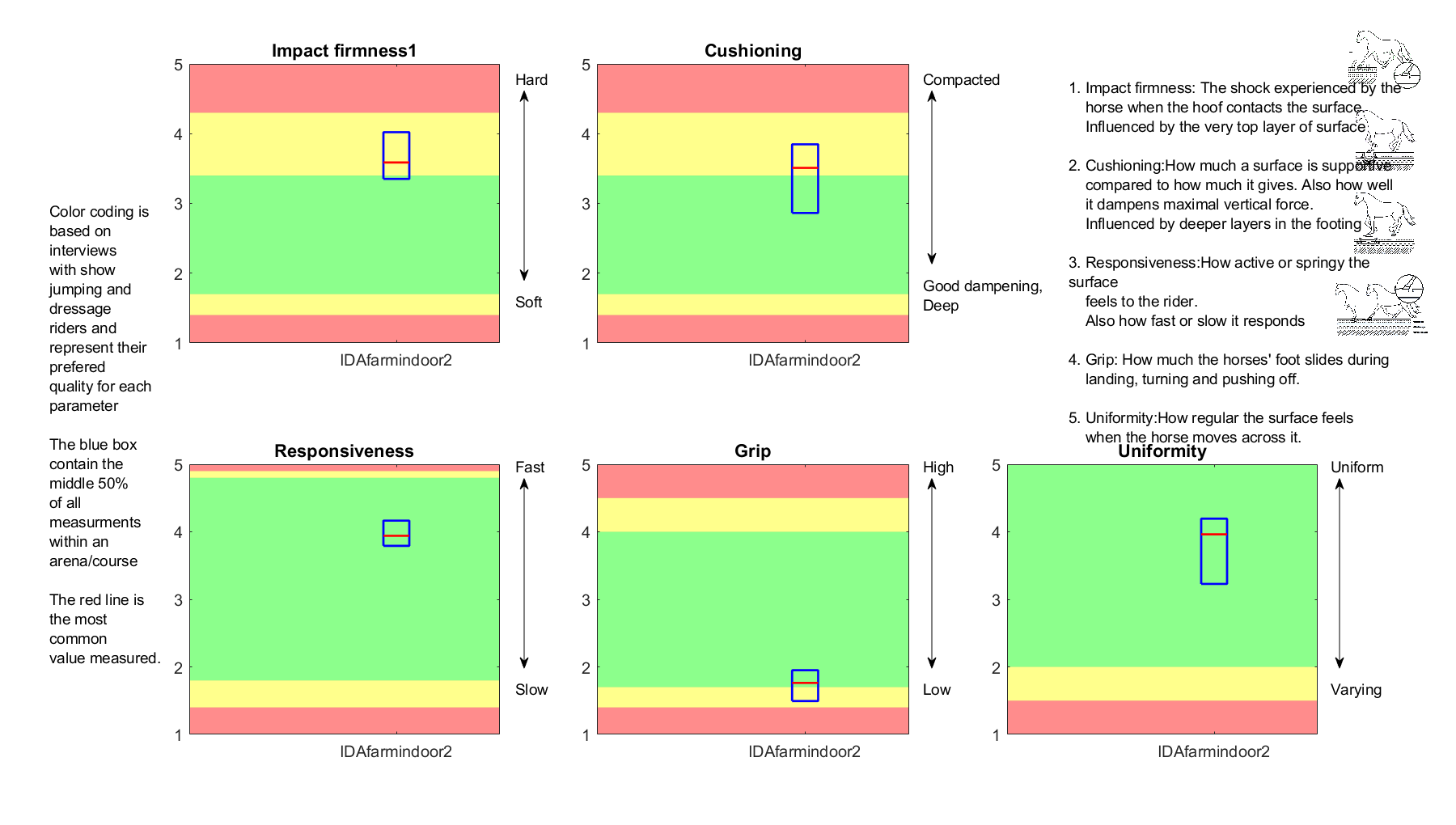


Figure 4b: The Orono biomechanical surface tester (OBST) showing the sand-fiber arena properties for impact firmness, cushioning, responsiveness, grip and uniformity. The OBST evaluation results graded as harder for impact firmness and more compact for cushioning.
